# Comparison of the effectiveness and safety of topical versus intravenous tranexamic acid in primary total knee arthroplasty: a meta-analysis of randomized controlled trials

**DOI:** 10.1186/s13018-017-0512-4

**Published:** 2017-01-19

**Authors:** Tao-ping Chen, Yu-min Chen, Jian-bao Jiao, Yun-fei Wang, Li-gang Qian, Zhao Guo, Zheng Ma, Cui-yu Han, Tong-huan Shi

**Affiliations:** 1 Affiliated Hospital of Hebei University, Yuhua Dong Road, 212, Yuhua District, Baoding, Hebei Province China; 2 Hebei University, Yuhua Dong Road, 342, Yuhua District, Baoding, Hebei Province 071000 China

**Keywords:** Tranexamic acid, Total knee arthroplasty, Meta-analysis

## Abstract

**Background:**

This study aims to compare the effectiveness and safety of topical versus intravenous tranexamic acid (TXA) in reducing blood loss in primary total knee arthroplasty (TKA).

**Methods:**

PubMed, Embase, the Cochrane Library, Web of Science, Chinese Biomedicine Literature (CBM), Wanfang Database and China National Knowledge Infrastructure (CNKI), and Google Scholar were searched for randomized controlled studies (RCTs) that compared topical versus intravenous TXA in terms of reducing blood loss during TKA from their inception to September 2015. This systematic review and meta-analysis was performed according to PRISMA criteria.

**Results:**

Twelve studies reporting 12 RCTs comprising 1130 patients were included. Compared with the intravenous administration of TXA, the topical administration of TXA showed no significant differences in total blood loss (MD 2.08, 95% CI −68.43 to 72.60, *P* = 0.95), blood loss in drainage (MD 18.49, 95% CI −40.01 to 76.98, *P* = 0.54), hidden blood loss (MD 4.75, 95% CI −337.94 to 347.44, *P* = 0.99), need for transfusion (RR = 0.92, 95% CI 0.67~1.25, *P* = 0.58), hemoglobin (Hb) decline (MD −0.42, 95% CI −0.89 to 0.05, *P* = 0.08), and DVT occurrence (RR = 1.17, 95% CI 0.55~2.50, *P* = 0.68).

**Conclusions:**

Compared with intravenous administration TXA, topical administration TXA exhibits comparable effectiveness and safety in terms of reducing blood loss during TKA. Due to the poor quality of the included studies, more high-quality RCTs are needed to identify the optimal method and dose of TXA after TKA.

## Background

Total knee arthroplasty (TKA) is an effective treatment that helps to relieve severe pain and handicap induced by knee joint disease. However, TKA may cause significant perioperative blood loss ranging from 800 to 1800 mL, and 10 to 38% of patients need allogeneic blood transfusion [1–5]. Blood loss and subsequent blood transfusion can increase cost, and many complications, such as infection with HIV or other infectious diseases, fluid overload, and graft-versus-host disease can occur [6].

Many methods have been used to decrease blood loss during TKA, including the use of tourniquet, fibrin sealant, and tranexamic acid (TXA) [7–9]. The hemostasis effect of tourniquet in TKA is controversial. Wang et al. [10] revealed that total and intra-operative blood losses were reduced only with long-duration tourniquet use, while Zhang et al. [11] found that tourniquet release at the end of the TKA will activate local fibrinolysis and might increase blood loss. Auguilera et al. [12] reported that TXA can be more effective than fibrin sealant in reducing postoperative bleeding and transfusion requirement and that fibrin sealant use was not superior to routine hemostasis.

The use of TXA in primary TKA is today widely accepted, and several studies and meta-analyses have confirmed the efficacy of TXA at decreasing blood loss without increasing complications and costs [9, 13, 14]. Several clinical trials have identified that intravenous (IV) TXA is effective in reducing perioperative blood loss and the need for subsequent blood transfusions in TKA [15–17]. However, deep venous thrombosis (DVT) after the systemic administration of TXA is still a serious and fatal complication [18]. Therefore, many researchers have focused on the topical application of TXA via drain tube or intra-articular administration, and the topical application of TXA is considered an alternative effective route that entails less risk than IV after TKA [19, 20]. A previous meta-analysis has been published that only included five RCTs and one non-RCT [21]. Nevertheless, the comparative efficacy and safety of topical versus intravenous TXA often differs between studies. Thus, we performed a meta-analysis to compare the effectiveness and safety of topical versus intravenous TXA after TKA.

## Methods

### Search strategy

The following electronic databases were searched: PubMed, Embase, the Cochrane Library, Web of Science, Chinese Biomedicine Literature (CBM), Wanfang Database and China National Knowledge Infrastructure (CNKI), and Google Scholar were searched by two reviewers. Relevant studies comparing the topical administration of TXA and intravenous TXA for the management of blood loss during TKA were identified by two reviewers; the search was performed in August 2015. The keywords and Medical Subject heading (MeSH) terms used for the search were the following: “TXA,” “tranexamic acid” “total knee arthroplasty,” “total knee replacement”, “TKA”, “TKR”, and “Arthroplasty, Replacement, Knee”[Mesh]; the terms were connected by the Boolean operators AND or OR. The search string used in our research is presented in appendix A. Additionally, the reference lists of all identified full-text articles were reviewed to identify any initially omitted studies. There were no restrictions regarding language. Since this is a meta-analysis, no ethics committee or institutional review board approval was required.

### Eligibility criteria and study quality

Study selection was performed according to the following inclusion criteria: (1) studies of patients undergoing primary TKA intervention; (2) with topical administration TXA and intravenous TXA as therapy to control blood loss; (3) studies assessing primary outcomes such as total blood loss, blood loss in drainage, hidden blood loss, need for transfusion, hemoglobin decline, and the rate of complications (DVT); and (4) studies designed as randomized controlled trials (RCTs). All the studies were required to be clinical trials. Trials on cadavers or artificial models were excluded. Letters, comments, editorials, and practice guidelines were also excluded.

According to the Cochrane Handbook for Systematic Reviews of Interventions (version 5.1.0) [22], Review Manager, version 5.3 (The Nordic Cochrane Centre, The Cochrane Collaboration, Copenhagen, 2014) was used to describe the quality assessment.

### Data extraction

Two reviewers independently extracted and recorded following data in a spreadsheet: (1) patient demographic data, author’s name, publication date, sample size, location of study, ratio of male and female subjects, the dose and method of TXA application (topical or intravenous), and whether TKA was unilateral or bilateral; (2) the method of anesthesia; and (3) total blood loss, blood loss in drainage, hidden blood loss, need for transfusion, hemoglobin decline and rate of complications (deep venous thrombosis (DVT)). If the data were presented as figures, the software “Getdata Graph Digitizer” was used to extract the data for meta-analysis.

### Outcome measures and statistical analysis

The main parameters studied were total blood loss, blood loss in drainage, hidden blood loss, need for transfusion, hemoglobin decline, and rate of complications (DVT). Hidden blood loss (mL) was calculated as total blood loss minus blood loss collected from drains. The results are presented as the mean differences (MDs) with 95% confidence interval (CI) for continuous outcomes such as total blood loss and blood loss in drainage. Binary outcomes, such as the need for transfusion and the occurrence of DVT were presented as relative risk (RR) values with 95% CI. Statistical significance was set at *P* < 0.05. To summarize findings across the trials, the software RevMan 5.3 (The Cochrane Collaboration, Oxford, UK) was used to conduct the meta-analysis. Statistical heterogeneity was tested using the chi-squared test and the *I* ^2^ statistic. Chi-squared test results with *P* > 0.1 were considered suggestive of statistical heterogeneity. An *I* ^2^ statistic greater than 50% was considered to indicate substantial heterogeneity [23]. A fixed effect model was adopted if the *I* ^2^ statistic value was less than 50%. If the *I* ^2^ statistic value was greater than 50%, a sensitivity analysis was performed using Stata 12.0 (Stata Corp., College Station, TX, USA) to explore the effect of an individual study.

## Results

### Search result and quality assessment

**Fig. 1:**
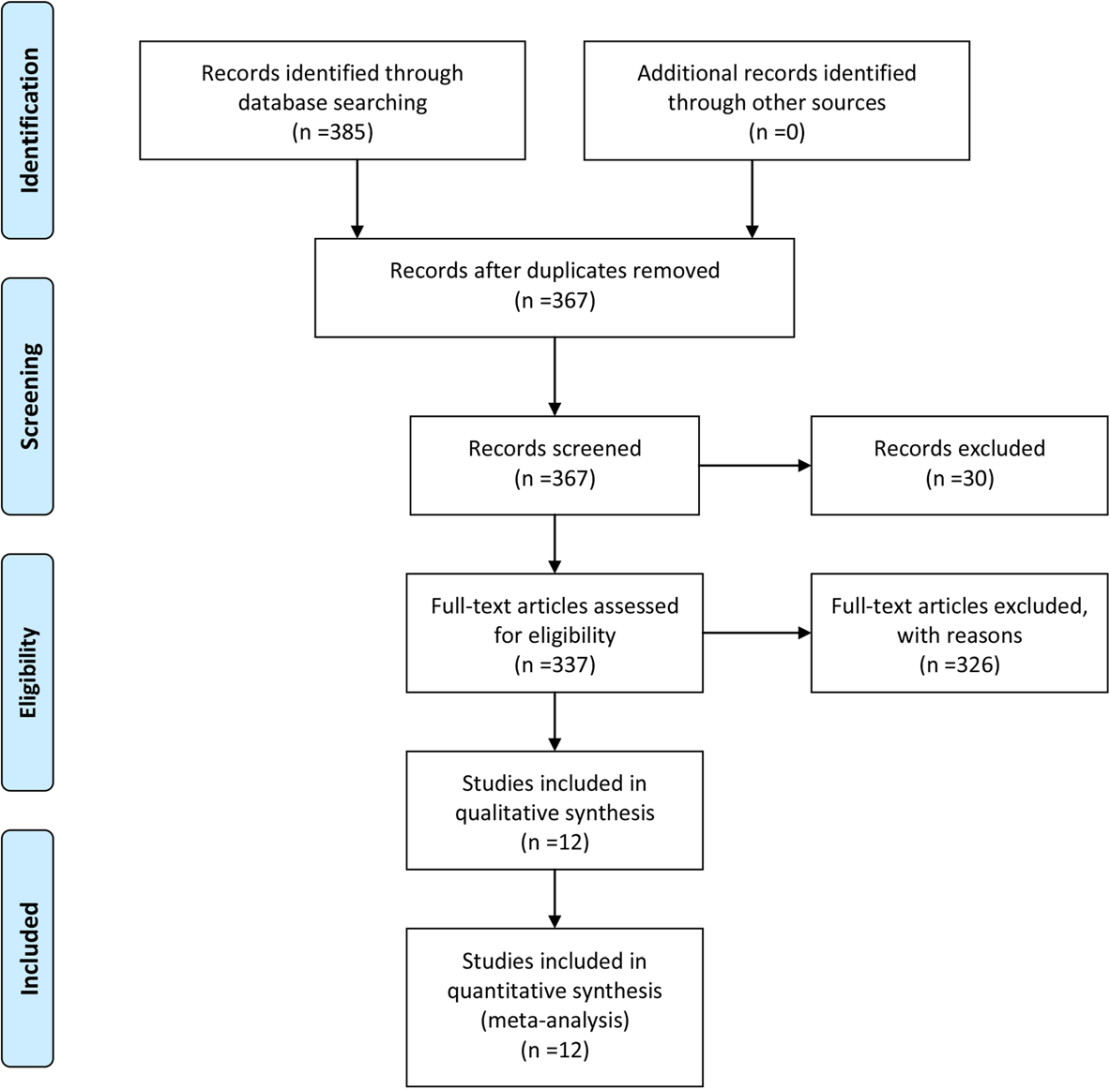
Flow diagram for the included studies

**Fig. 2:**
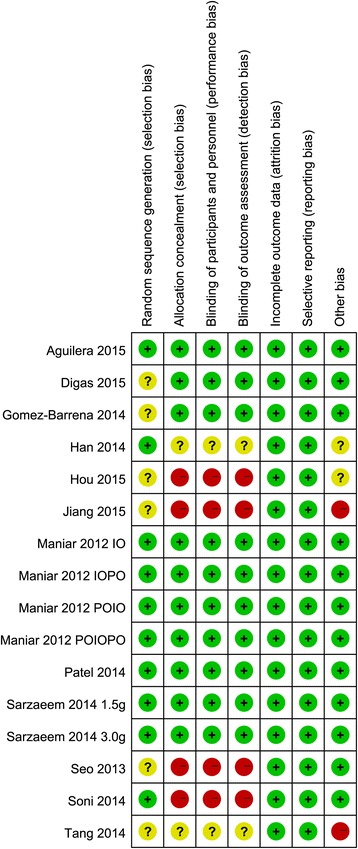
Summary of the risk of bias in each study

**Fig. 3:**
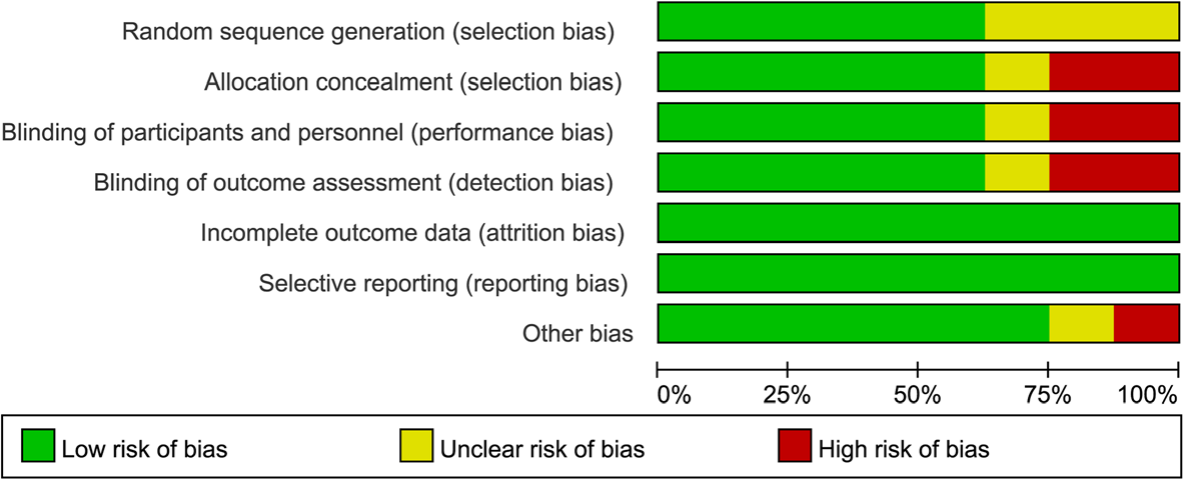
Graph of the risk of bias for the included studies

The initial search yielded 385 potentially relevant studies; of these, no duplicate was found using Endnote Software. Based on the inclusion criteria, 374 studies were excluded after reading the titles and abstracts. Finally, 12 clinical trials involving 1130 patients were included in the meta-analysis [24–36]. The process for selecting the included studies is presented in Fig. 1. One study included four IV groups [30], and one report included two topical groups that differed in the dose and timing administration of TXA [32]. Differently dosed TXA groups were included in this meta-analysis. The characteristics of the included studies are shown in Table 1, and the doses and methods used to apply TXA are summarized in Table 2. In the included studies, 1130 TKAs were performed, and the numbers of topical TXA and intravenous TXA were 529 and 601, respectively. Eight articles were in English, and four studies were in Chinese; all were published between 2013 and 2015. All participants in the 12 studies were elderly patients aged between 42 and 72.5 years who were planning to undergo TKA. Tourniquet was applied in all of the included studies, and the pressure of the tourniquet ranged from 100 to 350 mmHg. Detailed information regarding the risk of bias in the included studies is shown in Figs. 2 and 3.

**Table 1:** Characteristics of included studies

Authors	Patients (T/I)	Age (mean year) (C/I)	Gender (F/M)	Diagnosis	Country	Type of prosthesis	Surgical protocol	Pneumatic tourniquet	Follow-up
Patel JN 2014 [31]	47/42	42/64.9	23/66	OA	USA	ns	Con-TKA	Yes	4.6
Maniar RN 2012 [30]	40/160	67.4/67.45	42/158	OA	India	Cemented	CAS-TKA	Yes	3
Seo JG 2013 [33]	50/50	67.5/66.8	11/89	OA	Korea	Cemented	CAS-TKA	Yes	2
Sarzaeem MM 2014 [32]	100/50	67.8/66.9	20/130	OA	Iran	Cemented	Con-TKA	Yes	–
Soni A 2014 [34]	40/40	69.45/69.05	36/44	OA	India	Cemented	Con-TKA	Yes	1.5
Tang Lian 2014 [29]	30/30	66.5/67.1	20/40	OA	China	ns	Con-TKA	Yes	–
Han WF 2014 [26]	50/50	ns	20/80	OA + RA	China	Cemented	Con-TKA	Yes	3 months
Aguilera X 2015 [24]	50/50	72.53/72.49	70/30	OA + RA + DKD	Spain	Cemented	Con-TKA	Yes	2
Digas G 2015 [25]	30/30	70/71	51/49	OA	Germany	Cemented	Con-TKA	Yes	25 days
Jiang Hua 2015 [28]	33/40	ns	54/19	DKD	China	ns	Con-TKA	Yes	7 days
Hou ZY 2015 [36]	20/20	67.36/67.02		OA + RA	China	Uncemented	Con-TKA	Yes	14 days

*NS* not stated, *T* topical group, *I* intravenous group, *OA* osteoarthritis, *RA* rheumatoid arthritis, *DKD* degenerative knee disorders, *Con-TKA* conventional total knee arthroplasty, *CAS-TKA* computer-assisted total knee arthroplasty

**Table 2:** Dose and method for topical and intravenous administration of TXA

Author/s	Intervention		Methods of administration the tranexamic acid		Transfusion criteria	Thromboprophylaxis
	Topical	IV	Topical	IV		
Gomez-Barrenna 2014 [27]	3 g/100 mL	15 mg/kg. 2 doses	One dose irrigated before joint closure, and the other half was administered intra-articularly after skin closure	Fifteen to 20 min before tourniquet release and 100 mL 3 h	Hb <80 g/L	NS
Patel JN 2014 [31]	2 g TXA/ 100 mL NS	10 mg/kg ∗ 1 dose	2 min TA before tourniquet release, with drain	One dose as IO	Hb b 8.0 g/dL + symptom	LMWH
Maniar RN 2012 [30]	3 g TXA/100 mL NS	10 mg/kg ∗ 1 dose 10 mg/kg ∗ 2 doses 10 mg/kg ∗ 2 doses 10 mg/kg ∗ 3 doses	5 min TXA before tourniquet release, clamp drain 2 h then fully open	One dose as IO Two doses as IOPO Two doses as POIO Three doses at POIOPO	Hb b 8.5 g/dL Hb b 10.0 g/dL + symptom	LMWH
Seo JG 2013 [33]	1.5 g TXA/100 mL	1.5 g TXA/100 mL, NS	IXA while suturing, with no clamp drain	One dose at post-operative	Hb b 8.0 g/dL Hb b 10.0 g/dL + symptoms	NS
Sarzaeem MM 2014 [32]	1.5 gTXA/100 mL NS 3.0 gTXA/100 mL NS	1.5 g TXA/100 mL, NS	1.5 g TXA: IXA injected through the drain after wound closure 3 g TXA: 5 min TXA before suturing, clamp drain 1 h then fully open	One dose at post-operative	Hb b 7.0 g/dL Hb b 10.0 g/dL + symptom	NS
Soni A 2014 [34]	3 g TXA/100 mL	10 mg/kg ∗ 3 doses	5 min TXA before tourniquet release, clamp drain 2 h then fully open	Three doses as POIOPO	Hb b 8.0 g/dl	LMWH
Tang Lian 2014 [29]	500 mg/5 mL	10 mg/5 mL	Intra-articular injection after suction without drain	Intravenous 15 min before tourniquet release	Hb <90 g/L	LMWH
Han Wenfeng 2014 [26]	1 g/50 mL	15 mg/kg, maximum 1.2 g	Spray the TXA before suction and clamp drain for 30 min then fully open	Induction of general anesthesia	Hb <70 g/L	LMWH
Aguilera X 2015 [24]	1 g/10 mL	1 g * 2 doses	The TXA was applied by syringe spray and drain was kept closed during the first hour and removed 24 h after surgery	Patients in the control group received a slow IV infusion	Hb <80 g/L Hb <80 g/L + symptoms between 8.5 and 9 g/dL + symptoms	LMWH
Digas G 2015 [25]	2 g	15 mg/kg 1 dose	Intra-articular	Before deflation of the tourniquet	Hb <85 g/L Hb <95 g/L + symptoms	3.500 IU of tinzaparin
Jiang Hua 2015 [28]	2 g/100 mL	10 mg/kg 1 dose	Intra-articular	Before closing the wound	NS	Enoxaparin and Rivaroxaban
Hou zhenyang 2015 [36]	500 mg/10 mL	10 mg/kg 1 dose	Intra-articular	Before insert the prosthesis	Hb <70 g/L	Rivaroxaban

*NS* not stated, *Hb* hemoglobin, *LMWH* low molecular weight heparin, *IO* intra-operative, *IOPO* intra-operative and postoperative, *POIO* postoperative and intra-operative, *POIOPO* postoperative, intra-operative, and postoperative, *TXA* tranexamic acid

### Meta-analysis results

#### Blood loss (total blood loss, blood loss in drainage, and hidden blood loss)

**Fig. 4:**
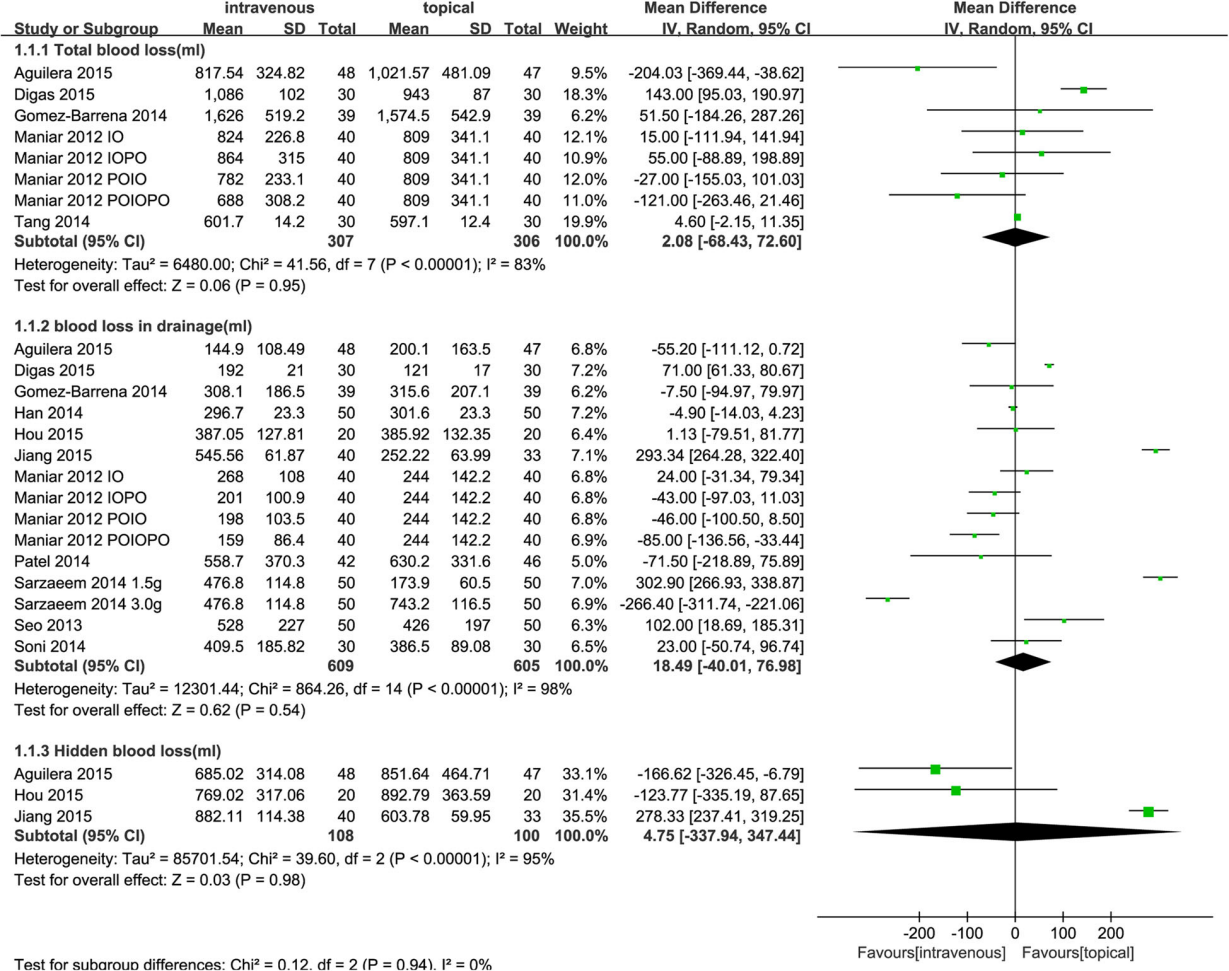
Forest plots comparing topical versus intravenous TXA administration regarding the need for transfusion. A Mantel-Haenszel fixed-effects model was used. Mean differences are shown with 95% CI

Five trials [12, 25, 27, 29, 30] reported data on topical versus intravenous administration for reducing total blood loss after TKA. Statistical heterogeneity was found (*χ* ^2^ = 41.56, *I* ^2^ = 83%, *P* < 0.00001, Fig. 4), and a random-effects model was performed. Topical administration of TXA showed no significant difference (MD2.08, 95% CI −68.43 to 72.60, *P* = 0.95, Fig. 4) in reducing total blood loss compared with intravenous TXA. A total of 10 studies [24–28, 32–34, 36] compared topical versus intravenous TXA in terms of reducing blood loss in drainage. Statistical heterogeneity (*χ* ^2^ = 864.26, *I* ^2^ = 98%, *P* < 0.00001, Fig. 4) occurred, and a random-effects model was performed. Similarly, the topical application of TXA did not significantly decreased the blood loss in drainage after TKA (MD18.49, 95% CI −40.01 to 76.98, *P* = 0.54, Fig. 4) compared with intravenous administration. For hidden blood loss, statistical heterogeneity (*χ* ^2^ = 39.60, *I* ^2^ = 95%, *P* < 0.00001, Fig. 4) was found, and a random-effects model was performed. There was no significant difference between the topical and intravenous groups (MD 4.75, 95% CI −337.94 to 347.44, *P* = 0.99, Fig. 4).

#### Need for transfusion

**Fig. 5:**
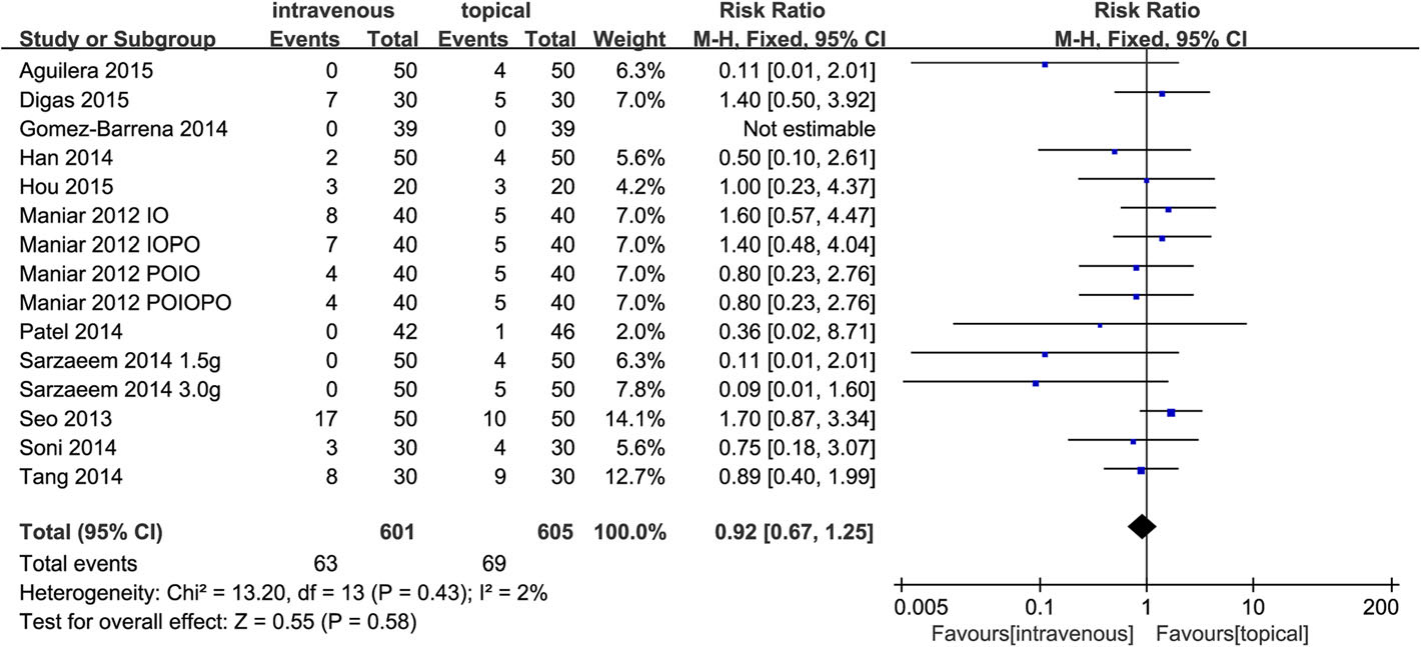
Forest plots comparing topical versus intravenous TXA for the need for total blood loss, blood loss in drainage, and hidden blood loss. An inverse-variance random-effects model was used. Mean differences are shown with 95% CI

Fifteen studies [24–27, 29–34, 36] mentioned a need for transfusion in 1206 enrolled knees. No significant heterogeneity (*χ* ^2^ = 13.20, *I* ^2^ = 2%, *P* = 0.43) was found. This paper uses a fixed-effects model, and no significant difference was found between topical TXA groups and intravenous TXA groups regarding the need for transfusion (RR = 0.92, 95% CI 0.67~1.25, *P* = 0.58) (Fig. 5).

#### Hemoglobin decline

**Fig. 6:**
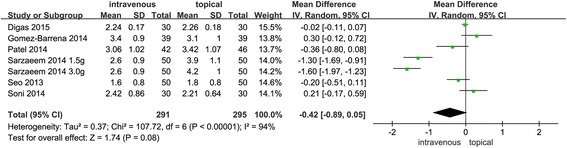
Forest plots comparing topical and intravenous TXA administration in terms of Hb decline. An inverse-variance random-effects model was used. Mean differences are shown with 95% CI

No significant difference was found between the IV TXA with topical TXA groups (MD-0.42, 95% CI −0.89 to 0.05, *P* = 0.08, Fig. 6). Statistical heterogeneity (*χ* ^2^ = 107.72, *I* ^2^ = 94%, *P* < 0.00001, Fig. 6) exists, and a random-effects model was performed. To eliminate heterogeneity, a subgroup analysis was carried out for hemoglobin decline (Table 3).

**Table 3:** Subgroup analysis for the hemoglobin drop

Outcome or subgroup	Studies	Effect estimate				
		*χ* ^2^	*I* ^2^(%)	MD	95% CI	*P* value
Non-computer navigated TKR	4	190.77	98	0.31	−1.26,1.89	0.7
High score RCT	3	193.26	97	0.11	−0.89,1.11	0.83

#### Thromboembolic complications

**Fig. 7:**
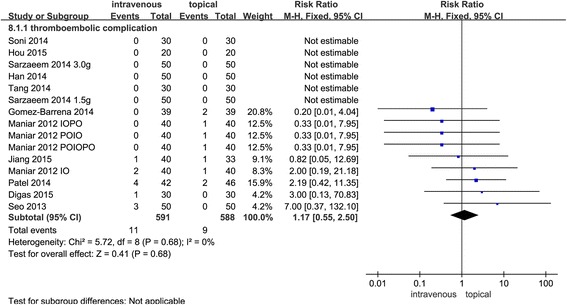
Forest plots comparing topical and intravenous TXA administration in terms of the occurrence of DVT. A Mantel-Haenszel fixed-effects model was used. Relative risk values are shown with 95% CI

Fourteen studies provided data about the rate of thromboembolic complications; the results of the meta-analysis indicate that the rate of thromboembolic complications for the intravenous group is 1.93% and that for the topical group is 1.58%; no significant difference was found between the two groups (RR = 1.17, 95% CI 0.55~2.50, *P* = 0.68, Fig. 7). No statistically heterogeneity (*χ* ^2^ = 5.72, *I* ^2^ = 0%, *P* = 0.68, Fig. 7) was found; therefore, a fixed-effects model was performed.

#### Other outcomes

Since the sample of other identified outcomes was not large enough for meta-analysis, we performed a descriptive analysis. For instance, Seo [33] found no significant difference between the range of motion between the two groups; the average ranges of motion (ROMs) at 2 months were 2.6°–123.3° and 2.5°–120.4° in the intravenous and intra-articular groups, and no significant difference was found in the intergroup analysis. The ranges of motion 1 month postoperatively were 104° ± 10° (95% CI, 101° to 108°) in the topical group and 105° ± 11 (95% CI, 101° to 109°) in the intravenous group (*P* = 0.612) [27]. There were 0 and 1 cases of atrial fibrillation in the topical TXA and IV TXA groups, respectively. Saezaeem [32] reported just one postoperative complication in the injected TXA group; this involved a patient with skin necrosis and more joint swelling than that seen for the intravenous group. One case of acute kidney injury was found in each of the topical and IV TXA groups [31].

#### Sensitivity analysis

**Fig. 8:**
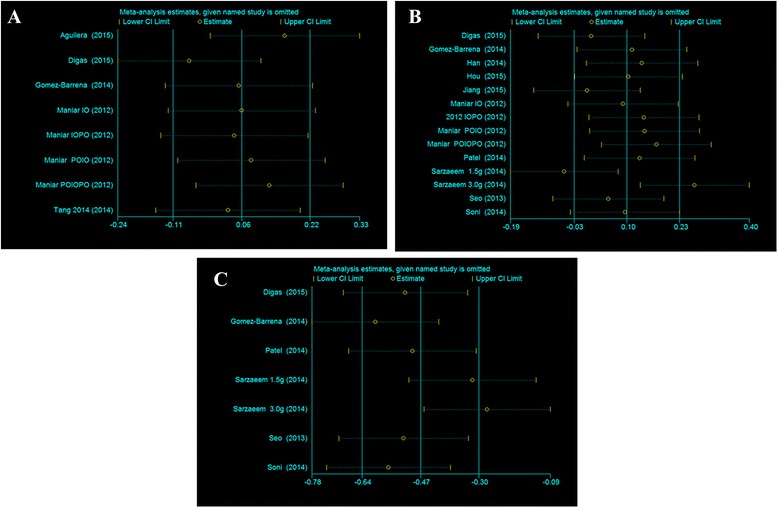
Sensitivity analysis of each end-point for total blood loss (**a**), blood loss in drainage (**b**) and hidden blood loss (**c**) after TKA

A sensitivity analysis of blood loss in drainage was conducted, and the results indicated that none of the studies affected the stability of the final results (Fig. 8).

## Discussion

The results of the current meta-analysis indicate that there were no significant differences in total blood loss, blood loss in drainage, hidden blood loss, need for transfusion, and minimal hemoglobin decline between topical TXA and IV TXA groups in primary TKA. Moreover, no significant difference was found in the rate of DVT between the two groups. Moreover, no adverse effects were reported in the included studies.

Patients undergoing TKA may suffer from large blood loss due to the large area of osteotomy and enhanced local fibrinolysis after tourniquet release [37]. Total blood loss can be 300 mL higher than that occurring with no tourniquet use. Thus, the topical administration of TXA can reduce postoperative blood loss when combined with the use of a drainage clamp. Furthermore, Jawhar et al. [38] reported that tourniquet use can induce ischemia during TKA and can result in higher proteolytic activity within vastus medialis cells, which may increase hidden blood loss. Compared with intravenous TXA administration, intra-articular TXA administration can be rapidly absorbed, maintaining a biological half-life of approximately 3 h within the joint fluid; this may be the reason why patients benefit from topical TXA administration [39]. TXA is an antifibrinolytic agent that activates plasminogen and stops bleeding without increasing plasma fibrin levels [40]. Several studies and meta-analyses have identified TXA as an efficacious and safe way to reduce blood loss in patients who undergo TKA [41–43], and numerous studies have focused on the efficacy of topical TXA administration in reaching a maximum hemostatic effect [44–46]. However, the most effective TXA regimen in primary TKA remains uncertain. A meta-analysis on outcomes was previously conducted to explore the difference between the two groups [21]. However, in their study, five RCTs and one prospective cohort study were included; moreover, they did not compare hidden blood loss between the two groups. The current meta-analysis included twelve studies and did compare hidden blood loss, which comprises a large proportion of total blood loss.

With regard to the need for transfusion, no significant difference was found between the two groups (RR = 0.92, 95% CI 0.67~1.25, *P* = 0.58); the dose of topical administration ranged from 0.5 to 3 g, and the dose of intravenous TXA administration ranged from 10 to 15 mg/kg. Three studies reported hidden blood loss for the two groups, and Aguilera [24] favors the use of topical administration to reduce hidden blood loss after TKA, whereas Hou and colleagues [47] found no difference between topical and intravenous administration. In this meta-analysis, no significant difference between topical and IV TXA administration was found. Our sensitivity analysis showed that the study of Sarzaeem [32] significantly affected the heterogeneity results. After carefully reviewing the study, the main difference between this study and the other studies is that TXA was administered by irrigating with 3 g of TXA and injecting with 1.5 g TXA. In addition, doses of topically administered TXA ranging from 0.5 g [48] to 3 g [27, 30, 34, 44, 49] have all proven effective and safe. Regarding blood loss in drainage, a large degree of heterogeneity was found between the two groups, and the sensitivity analysis showed that the inclusion of Sarzaeem’s study influences the final conclusion. After carefully reading the article, the topical administration of TXA was applied by irrigation.

DVT and subsequent PE are fatal complications after TKA, and it has been reported that 0.1–2% of patients will suffer from pulmonary embolism following TKA [50]. Theoretically, intravenous TXA administration may increase the risk of thrombosis since it is a synthetic antifibrinolytic agent that can prevent plasminogen activation, and fibrinolysis is delayed [51]. However, it has been ascertained that TXA does not inhibit fibrinolytic activity in the vein wall, and its effect on disrupted endothelium remains unknown [52]. The results of the current meta-analysis indicate that both topical and intravenous administration TXA are safe when used to reduce blood loss during TKA. The frequencies of DVT after intravenous and topical TXA administration are 11/571 and 9/568, respectively (RR = 1.17, 95%CI 0.55~2.50, *P* = 0.68). The reasons for this may be as follows: (1) the doses used during intravenous and topical administrations are appropriate and do not exceed the optimal dose; therefore, the fibrinolytic system will not be suppressed. (2) Most studies only evaluated symptomatic DVT and PE, and this will have affected the final conclusion.

This meta-analysis has the following limitations: (1) Only twelve studies were included, and the sample sizes used in each study were insufficient, which will affect the conclusions. (2) The studies administered TXA at different doses and using different methods, causing large heterogeneity. (3) The duration of follow-up in some studies was short; therefore, long-term follow-up is needed to measure the occurrence of DVT and PE.

## Conclusions

In conclusion, this meta-analysis indicated that topical TXA administration exhibits comparable efficacy and safety to those of intravenous TXA, and both methods can decrease the need for transfusion, total blood loss, blood loss in drainage, and hidden blood loss without increasing the occurrence of DVT. Due to the poor quality of the included studies, more high-quality RCTs are needed to identify the optimal method and dose of TXA after TKA.

## Abbreviations

CBM: Chinese Biomedicine Literature
CI: Confidence interval
CNKI: China National Knowledge Infrastructure
DVT: Deep venous thrombosis
IV: Intravenous
MD: Mean differences
Mesh: Medical subject heading
PRISMA: Preferred Reporting Items for Systematic Reviews and Meta-Analyses
RCTs: Randomized controlled trials
RR: Relative risk
TKA: Total knee arthroplasty
TXA: Tranexamic acid
